# Nuances of balance; parental perspectives around screen use and nature exposure in middle childhood

**DOI:** 10.1186/s40359-025-03552-9

**Published:** 2025-12-22

**Authors:** Marina Torjinski, Sharon Horwood

**Affiliations:** 1 ARC Centre of Excellence for the Digital Child, Queensland University of Technology, Brisbane, Australia; 2 School of Psychology, Deakin University, Locked Bag 20000, Geelong, 3220 Australia

**Keywords:** Screen Use, Parent-Child relations, Green space, Nature therapy, Children, Family leisure

## Abstract

**Background:**

Limitations of existing screen guidelines combined with the prevalence of parental concerns around children’s screen use necessitate the need for novel approaches to screen use management that are family-centred and focus on health-positive rather than restrictive messaging. A health-promoting activity that is inversely associated with excessive child screen use across numerous psychosocial outcomes and may lead to greater improvements in family functioning compared to other activities, is nature exposure. However, the joint and reciprocal relationships between child screen use and nature exposure have not been investigated in the relational family context.

**Methods:**

Qualitative data was collected from 20 primary caregivers of children aged 5–8 who participated in semi-structured online interviews. A template thematic analysis comprising both deductive (conceptual and definitional scope for coding was determined through an a priori template) and inductive processes guided theme generation.

**Results:**

Three main themes were generated, representing commonalities across parent accounts and intersections between key research areas: (1) ‘fixation, obsession and displacement’, (2) ‘is screen use facilitating opportunities or driving disconnection?’ and (3) ‘parental self-efficacy is fluid’. The findings reveal meaningful interactions between children’s screen use and outdoor time, that map onto the interactional theory of childhood problematic media use. We propose family-based nature activities as an additional protective factor for the etiology of problematic child media use.

**Conclusion:**

Findings provide a more integrated and nuanced understanding of how children’s screen use and engagement with nature interact within the family paradigm. This research may help generate evidence-based alternatives and practical approaches to help parents navigate some of the challenges associated with childhood screen use.

**Supplementary Information:**

The online version contains supplementary material available at 10.1186/s40359-025-03552-9.

Screen-based technologies such as smartphones and tablets are becoming an increasingly central part of modern family life, transforming family routines, processes, and interactions. While there are many positive uses for digital technology (e.g., knowledge acquisition and social connectivity), there is growing evidence of an association between excessive child screen use and adverse physical, psychological and behavioral health outcomes [1, 2]. Parents face significant challenges in meeting rigid screen time guidelines and maintaining healthy digital family practices [3] and almost 60% of school-aged children globally exceed recommended amounts of screen time (typically no more than two hours per day for children aged 5 to 12) [4]. While current screen time guidelines focus on restricting children’s access to screens [5, 6], evidence-based recommendations suggest a shift toward parent-centered interventions that emphasize balancing screen time with other health-promoting behaviors [7]. One health-promoting activity that could be an effective screen management strategy is time spent in nature. In addition to the well-known physical health benefits of outdoor play, environments characterized by features of nature uniquely promote wellbeing across various psychosocial dimensions [8]. Importantly, natural environments appear to inversely influence psychosocial wellbeing outcomes associated with excessive child screen use [9]. For example, while excessive screen use may deplete cognitive resources and increase levels of stress [10], natural settings can promote reductions in stress [11] and lead to improvements in mood [12] and various cognitive functions [13]. Furthermore, family-based nature activities may promote both child and parent-level wellbeing outcomes that in turn could facilitate positive shifts in relational dynamics associated with children’s screen use. Importantly, fostering children’s connection with nature through positive family-based nature activities may promote value systems that lead to long-lasting preferences for health-promoting activities. At present, the joint and reciprocal relationships between children’s screen use and exposure to nature, and related family-level mechanisms such as parental management of these child health behaviors, remain under-investigated [14]. Therefore, a qualitative investigation with a focus on parental perceptions is required to contextualize the interplay of child routines around nature and screen use within a relational family paradigm.

## Problematic screen use in the family context

Although conceptualizations of middle childhood vary across the literature, the ages between 5 and 8 represent a sensitive developmental period characterized by increasing autonomy and steep developments in children’s socioemotional competencies [15]. During this period, parents play a key role in scaffolding children’s daily activities and health behaviors, while also influencing their health-related beliefs through complex dyadic interactions [16]. Thus, it is important for child screen use research to explore the role that parents play in bidirectionally influencing children’s screen-related attitudes and behaviors during this developmental stage. Research has identified numerous parent and family-level correlates to children’s screen use including physical home environment, parental screen beliefs, and parental screen-related self-efficacy (a parent's perceived capability to effectively navigate screen-related challenges) [17]. However, there is limited understanding around how child-, parent-, and family-level factors (e.g., child temperament and parental stress) interact with each other, and with broader socio-environmental factors (e.g., digital environment) and developmental influences (e.g., other health-promoting activities) to shape children’s screen-related outcomes.

The etiology and maintenance of problematic child screen use (often termed excessive screen use) appears to involve a complex, dynamic, and interactional set of factors. Domoff, Borgan, and Radesky [18] define problematic childhood media use as patterns of maladaptive behaviors characterized by excessive use of digital media that interfere with a child’s functioning. On an individual level, behavioral manifestations of problematic child screen use may include difficulties with emotional regulation, pre-occupation with screen media, and loss of interest for other activities, as well as deceptive screen-related behaviors [18]. On a relational level, problematic behaviors may interfere with dyadic parent-child processes such as communication and co-regulation. The interactional theory of childhood problematic media use [18] illustrates some of the complex mechanisms through which immediate factors (e.g., child characteristics and family dynamics) interact with distal systems (e.g., digital environment) to influence children’s screen use behaviors. For example, persuasive features of the digital environment (e.g., curated content and fast-paced flashing graphics) may influence the interaction between screen-related child behaviors and parental stress resulting in relational conflicts that further perpetuate maladaptive patterns of screen use [18, 19]. Although prior research has provided tentative evidence of explanatory problematic screen use pathways, interactions between within-family processes and other contextual factors associated with children’s screen use require more nuanced examination. Furthermore, a greater understanding of how multiple determinants of child health interact with factors that either protect against or maintain maladaptive patterns of child screen use is needed.

### Challenges and opportunities for parental screen management

Despite growing parental concern around the potential influence of screen use on child health and development [20], parents often do not meet their own ideal limits regarding their children’s screen use [3, 21]. Likewise, interventions focused on parental media awareness have not consistently led to effective parental screen limiting practices [22].

Qualitative evaluations of health-focused family interventions revealed that parents hold unfavorable views around negatively framed screen time messaging [23], which are a common source of parental guilt [3, 23], and consequently challenge parental self-efficacy beliefs [3]. Parental self-efficacy relates to a parents perceived ability to carry out effective parenting strategies in the face of associated challenges, both broadly (general parental self-efficacy) and in relation to specific parenting tasks (domain-specific parental self-efficacy) [24]. While low parental self-efficacy for managing screen time is a significant predictor of increased screen time in children [19, 25], parents with higher screen-related self-efficacy are more likely to engage in healthy screen limiting practices [26].

Compared to restrictive screen-related messaging, parental messaging that reinforces health-positive child behaviors, such as physical activity, is associated with higher levels of perceived parental self-efficacy [27]. Additionally, family-based interventions that focus on mutually beneficial and enjoyable family routines are more likely to foster ongoing child engagement in health-positive behaviors [23]. Thus, it may be more beneficial to influence screen-related child and family outcomes indirectly through approaches that focus on positive parent-child interactions and health-promoting family routines that increase parental self-efficacy. However, such approaches have been largely overlooked by existing screen-awareness campaigns [23] and there is limited understanding of what strategies can effectively empower parents with the confidence to manage screen-related challenges in real-life contexts.

### Family-based nature activities, wellbeing, and parental self-efficacy

Although general outdoor time is associated with positive health outcomes for children and adults, time in nature-rich environments such as tree-lined parks and nature reserves, offers unique benefits to psychosocial wellbeing [8]. Specifically, natural environments offer rich opportunities for families to develop health-promoting routines that have the potential to facilitate improved family wellbeing and parental self-efficacy [28]. The theoretical basis for this lies in Attention Restoration Theory [29], the idea that natural settings promote individual wellbeing through restoration of mental processes that become fatigued from tasks requiring directed attention (such as prolonged screen use). In addition to the unique benefits of direct contact with nature such as improved mood, cognitive function, and reduced stress [8], perceived nature connectedness and shared symbolic meanings that families attribute to nature-based activities are associated with increased parent and child wellbeing [30]. Izenstark and Ebata’s integrative model of family-based nature activities [31] proposes that family routines and ritualized nature-based activities facilitate positive family processes through a combination of direct nature experiences and perceived nature connectedness. Due to the restorative effects of nature on mental wellbeing, the authors propose that nature-based activities promote more positive family interactions compared to other leisure activities. In testing their model, they demonstrated that compared to indoor walks, parent-child walks in nature improved individual mood and attention restoration, facilitated dyadic cohesion, and resulted in more positive family interactions [32, 33]. Other research has demonstrated that following positive family experiences in nature, parents reported increases in parental self-efficacy around family engagement with outdoor activities [34, 35].

### Current study purpose and aims

Despite the relevance of this topic to related fields of research, a recent review indicates that few studies have looked at the combined influence of children’s screen use and nature exposure in the family context [14]. Considering both the cultural variations and between-family variables associated with family lifestyle behaviors, a deeper examination of how families conceptualize, perceive, and engage with nature-based and screen-based experiences is needed to understand how these health behaviors interact with contextual nuances and relational outcomes. Accordingly, the current qualitative study aimed to investigate the mechanisms through which family routines around nature and screen use interact with the relational family system. Reflecting the broad, open ended, and exploratory research aim, the study seeks to answer the following question: How do family routines around nature and screen use interact with family dynamics, from parents’ perspectives? A more integrated understanding of how children’s screen use interacts with other upstream determinants of health such as nature exposure could help identify novel and modifiable targets for family-based intervention. This may inform development of practical solutions for parental screen management as well as effective delivery of public health messaging.

## Methods

### Methodological approach

The present study followed a qualitative research design to explore parental perceptions around family routines involving children’s screen use and nature exposure. Research was carried out between July and September 2023, across Australia. Ethics approval for the study was received from the Deakin University Human Research Ethics Committee (HEAG-H 49_2023). This research adopted a relativist perspective within a constructivist epistemological paradigm, which views reality as subjective and shaped by unique individual and sociocultural contexts. From this perspective, both participants and researchers are co-creators of knowledge and meaning. Accordingly, we examined the multiple and interrelated factors shaping parents’ lived experiences and perceptions while critically reflecting on our own perspectives as researchers. Our research team (one parent [MT] and one non-parent [SH]) recognizes the value of parents’ rich lived experiences in providing nuanced perspectives around family processes, dynamics, and interactions. As researchers within the field of psychology, we have adopted a broad view of health which explores how biopsychosocial systems interact to influence human perception, decision-making, and behavior. By contextualising our research within the relational family paradigm, we acknowledge the bidirectional and symbiotic relationships between child, parent, and family wellbeing.

### Participants and recruitment

Participants were parents of children aged 5–8, recruited through an optional extension of an online survey for a larger study investigating associations between children’s screen use, nature exposure, and the parent-child relationship. Participants were recruited to the original survey through numerous online methods including social media (e.g., Facebook [the authors’ institutional pages and popular parenting groups], Instagram, Twitter), as well as email (organizers of popular parenting forums and groups, child-focused associations, and primary schools) and media exposure (print, online, radio, and television broadcasting). Verbal, online, and email snowballing was also employed (e.g., participants asked if they would like to share the survey link upon survey completion).

A purposive sampling matrix (see Online Resource 1) guided the selection of participants from the larger pool of survey respondents who indicated their interest in participating. Demographic data for the participating parent and their nominated child was available from the previous study, along with baseline data relating to family engagement with screen devices. To represent a variety of parental perceptions, family characteristics and experiences, the purposive sampling matrix considered a range of demographic factors (age of parent and focus child, household income, education, employment, and family composition), as well as variation between children’s screen time. Of the 24 participants who provided informed consent to being interviewed, four did not respond to further contact.

The resulting sample consisted of 20 English-speaking Australian parents (18 Females, 2 Males, ranging in age from 28 to 49). Although the gender, age, and screen time of participants’ focus children was distributed evenly, most participants were women (90%), received a higher tertiary education (85%), and were employed (90% [part-time, full-time or casual]). Complete sample characteristics can be viewed in Online Resource 1. The resulting sample size was agreed upon by the research team in consideration of sample breadth (maximum diversity and variation between available participant characteristics) and opportunity to obtain rich, in-depth individual case data. Although participation was voluntary, participants were offered a $30 Coles Voucher as a token of appreciation for their time.

### Procedure and analysis

Participation involved semi-structured one-on-one interviews conducted through the Zoom^tm^ online platform by Author 1(MT) and two student interviewers. Interview times ranged from 19 to 55 min (average duration of 40 min) and were auto-transcribed and corrected manually to ensure verbatim response data. Questions were primarily open ended, with prompts to stimulate further elaborations or clarifications. Sequencing of questions was established by the research team to reduce potential response bias (see Online Resource 2) and to explore the perceived role of specific research topics within broader family contexts. For example, more general questions such as ‘what does quality family time look like to you’ were asked first, and more specific questions such as ‘how often does your child request to use screen devices when you’re out in nature’ were asked afterwards. Care was taken to ensure that researcher responses did not suggest personal value judgments.

A template analysis approach [36] was adopted, to allow for flexibility between deductive and inductive processes of analysis. This approach is a subtype of qualitative thematic analysis that allows researchers to develop theory-driven a priori categories to guide and organize coding of relevant data and explore patterns across the data to generate more nuanced thematic relationships [37]. Such flexibility in qualitative analysis is particularly important for explorative research synthesizing conceptually novel, interdisciplinary research areas.

For the present study, all textual data were processed and coded through NVivo V.12 software. Following a systematic scoping review of thematically relevant literature conducted by the authors [14], an a priori coding template (see Online Resource 3) was collaboratively developed. Due to the novel intersection of research areas, the a priori template functioned as a framework for the deductive organization of data related to each study topic (screen use, nature exposure, and relational family dynamics) based on existing research and theory. Specifically, the coding template defined the scope for coding, including conceptual and definitional areas of focus. Although the a priori template was informed by prior research conducted by both authors, initial coding was undertaken by the principal investigator. This process involved the formation of ‘nodes’ - units of textual data representing coding template categories. After initial coding of a subset of five transcripts, the coding template was refined by Author 1, and the remaining data were coded through a recursive process informed by regular discussions between the research team.

The present study aimed to identify and explore joint and reciprocal relationships between key research areas. Therefore, rather than following the a priori template structure to organise themes and subthemes, a new thematic map was created using an inductive analytic approach informed by frequency of patterns across the data as well as areas of thematic intersection representing conceptually rich data relevant to the research question. Coding categories, themes and subthemes were discussed by members of the research team through online meetings to stimulate critical reflection around meaningful thematic relationships (as recommended by Brooks and King [38]). Visual mind-mapping and a shared virtual spreadsheet were used to assist reflexive development and refinement of thematic categories and relationships until consensus was reached by the research team.

## Results

**Fig. 1:**
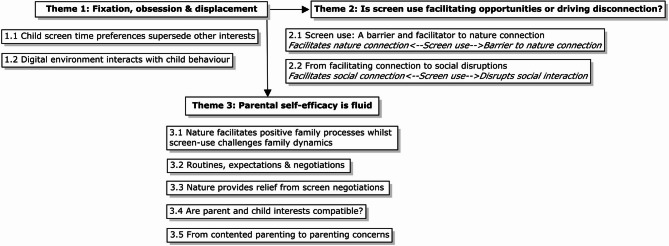
Thematic Relationships

This qualitative study was designed with a transdisciplinary focus, exploring areas of overlap between the research domains of screen use, nature exposure, and relational family wellbeing. Accordingly, themes and subthemes reflect areas of intersection between these domains. Data collected from parent interviews was classified into three themes: (1) ‘fixation, obsession and displacement’, (2) ‘is screen use facilitating opportunities or driving disconnection’? and (3) ‘parental self-efficacy is fluid’. Each theme was then divided into subthemes, as shown in Fig. 1. Theme 1 explores parental perspectives around how children’s screen use interacts with features of the digital environment, child and parent-level factors, and family wellbeing. Themes 2 and 3 are conceptualized as thematic continuums, with subthemes illustrating the fluid junctures between key research areas which are sensitive to unique child, parent, and family-level characteristics as well as wider social and environmental contexts.

### Theme 1: fixation, obsession and displacement

Although parents generally held balanced views around children’s screen use and acknowledged the positive uses of technology within the home and society, parental concerns around the influence of screen use on child and family wellbeing predominated the data. Furthermore, dimensions of these screen-related concerns wove in meaningful ways throughout the data, reportedly interacting with other aspects of parental decision-making, perceived child behavior, family dynamics, and routines. Following a recursive process of collapsing and refining themes and subthemes, a decision was made to represent screen-related parental concerns as a freestanding theme. Accordingly, Theme 1 summarizes the most prevalent commonalities across parental accounts, that also interact with and contextualize relationships across themes 2 and 3 (as illustrated in Fig. 1).

### Child screen time preferences supersede other interests

A common challenge reported by parents was knowing how to integrate screen time into the family routine while retaining a sense of balance: “I need some support on how I can integrate it into our lives without it becoming something that overtakes his behavior” (*Mother of a 5yo*,* 41yo*,* 3 children*). Parents believed that without parental regulation, children would be on devices “all day” and “drop everything” (*Mother of a 6yo*,* 42*,* 2 children*) due to a preference for screen time over all other activities. Hence, parents were concerned about screen activities superseding children’s interest in face-to-face, hands-on, and creative play: “They go from having beautiful creative times when there’s no screens… as soon as there’s a screen available, that just totally overrides any other play” *(Mother of a 7yo*,* 43*,* 4 children).*

### Digital environment interacts with child behavior

A common concern expressed by parents, was the perceived influence of screen use on child behavior. These beliefs were influenced by their own prior experiences, as well as social observations where children’s problematic screen-related behaviors “cause the most issues” (*Mother of a 5yo*,* 33yo*,* 3 children*) for other families. Problematic behaviors reported by parents included dysregulated reactions to screen time rules such as “tantrums” and “outbursts”, frequent and persistent screen time requests, difficult transitions away from screen time, and deceitful behaviors. One parent described how her child “wasn’t able to stop” when engaged in screen-based activities, leading to interference in bodily functioning: “he ended up wetting himself and having accidents… even though he needed to eat or go to the toilet… he just couldn’t control that stopping” (*Mother of a 5yo*,* 41yo*,* 3 children*). For many parents, difficulties navigating problematic screen-related child behaviors led them to “consciously avoid” (*Mother of a 5yo*,* 33yo*,* 3 children*) screen time altogether.

Parents also discussed how child traits and dispositions interact with screen-related inclinations and behaviors, ultimately influencing parental decision-making around provision of screens. Parents who described their children as “easily attracted” to screens, reported that even small amounts of screen time result in difficult behaviors. Other children with “easy going” temperaments were perceived as better able to regulate their use and less resistant to screen time rules. Several parents highlighted these differences through sibling comparisons:

It’s different between the kids. Ben, I could have the TV on all day, he might watch some of it, he’ll be playing, he might watch a bit more again- but it doesn’t attract his whole attention… But Tom, even from a young age, could be very focused on it, and do nothing else. So, for him, we’re turning it off because otherwise, it’s too much. (*Mother of a 5yo*,* 38*,* 2 children*)

Terms such as “obsessed”, “fixated” and “consumed” were frequently used by parents when describing their children’s screen use, such states are often attributed to the persuasive features and curated screen content targeted at young children: “it’s designed, it’s curated, it’s directed in a way to capture the audience… I don’t think it’s evil or sinister, it’s just effective… it draws children in, it knows how to make them keep watching” *(Father of a 5yo*,* 41yo*,* 2 children)*. Parents described screen time as addictive: “The more TV he gets, the more he wants… you could definitely see that kind of addictive stimulation coming through even after a couple of hours, which is a reminder of how powerful it is” (*Mother of a 6yo*,* 38yo*,* 1 child*). According to parents, the combination of children’s inability to regulate their screen use, combined with persuasive elements of the digital environment, often resulted in “conflict”, “frustration”, and an arduous demand of parents’ energy.

### Theme 2: is screen use facilitating opportunities or driving disconnection?

Captured within continuums between connection and disconnection, this theme explores parental perspectives around how and why screen use can both strengthen and challenge children’s relationships with other people and the natural environment.

### Screen use: a barrier and facilitator to nature connection

The way parents conceptualized the relationship between children’s screen use and engagement with nature varied across unique family dynamics and screen-contexts. Many parents reported that screen time was “not an option” (*Mother of an 8yo*,* 44yo*,* 2 children*) for children during nature visits, however it was also common for parents to report on the practical utility (e.g., safety) and educational benefits of technology during nature-based activities. They described a range of screen uses in nature such as taking photos, “mapping and distance measuring” *(Mother of a 6yo *,*47yo*,* 1 child)* and finding information: “sometimes they ask me a question, and I have got to look, because I don’t have all the information… like learning the names of the birds and the fish and all this kind of thing” *(Mother of a 7yo*,* 41yo*,* 2 children)*. At times, parents held contrasting views between general screen use and screen-based activities that directly facilitated children’s nature connection:

“If we’re out doing something, and she’s like, oh, what’s that mum… we might take a photo of a flower, and use Google Lens to look it up and see what the tree is, or a random animal is… but other than that - no, we don’t [use screen-based devices]”.

Reflecting on her own experiences as a parent and the broader challenges faced by families of neurodivergent children, one parent discussed the benefits of screen use as a strategy for her child to connect with nature by overcoming associated barriers:

We always take his accessibility devices, it allows him to access nature. I think a really important consideration in this whole conversation is that it doesn’t have to be screen time or green time, we often take our screens into nature. (*Mother of a 6yo diagnosed with ASD*,* 36yo*,* 4 children*)

While parents identified the positive uses of technology for nature connection (contingent upon screen use context and content), children’s screen use was more broadly perceived as a barrier to nature connection. Perceived mechanisms included the influence of peer networks that promote screen-based over nature-based activities, and children’s preoccupations with screen-based activities interfering with other free-time interests (as discussed in theme 1.1).

### From facilitating connection to social disruptions

Variations across parent accounts provide examples of how screen-based technology can both disrupt social interactions, and connect children with family members and broader social networks. Parents commonly reported that children’s screen-based preferences drove them to choose solo, sedentary screen time over other types of interactive “face-to-face” activities. Some participants expressed the belief that a reliance on technology during social interactions can disrupt relational opportunities. These perceptions were informed by parents' own experiences as well as social observations: “They’re not talking, they’re not interacting, they’re not sharing anything with each other. They’re all just scrolling on their phones, and I don’t want that for him [participant’s son]” (*Mother of a 7yo*,* 48yo*,* 1 child*). Some participants’ described the use of screens to distract and occupy children during social experiences as lost opportunities for face-to-face interaction, communication, and co-regulation:

For our family, those little moments where you could use them [screens] to take the pressure off is where we do get the connection… I take the kids grocery shopping every week and they sit in the trolley and we talk about what we’re getting and we play funny games… that’s now a time for connection as opposed to - let me just get this done and you can watch something… sometimes they do have a little moment in the shops, or they start pushing each other in the trolley… but that’s all part of learning… that’s a moment that they get to learn about delayed gratification or about getting along together or something like that and they’re moments that we wouldn’t have got to have if we had been distracted by something else. (*Mother of a 5yo*,* 33yo*,* 3 children*)

However, parents simultaneously held positive views around specific screen applications and uses facilitating social connection. This included bonding over program and movie co-viewing or shared enjoyment of video games. The combination of connection and educational content was particularly valued by parents: “she loves playing Roblox at the moment… she really enjoys the networking… talking to her friends on there. But it’s also really creative” (*Mother of a 7yo*,* 49yo*,* 1 child*). One parent provided an example of her son engaging in cultural learning in a video call with extended family:

We’re not living on his country right now, so it allows him to have constant connection with his cousins and his uncles… he games with his uncles, he learns lots of language and amazing things by just talking to his uncles every day. (*Mother of a first nations 6yo*,* 36yo*,* 4 children*)

### Theme 3: parental self-efficacy is fluid

This theme illustrates the multifaceted ways in which individual, relational and environmental factors interact with parenting beliefs and motivations.

### Nature facilitates positive family processes while screen use challenges family dynamics

When discussing the interaction between family routines and relational family dynamics, children’s screen use and nature exposure were often described as separate, distinct constructs. However, the relationships between these activities, relational family processes, and parental self-efficacy perceptions were generally framed in contrasting ways. Nature-based family activities such as gardening, camping trips, park and beach visits were described as “quality time” and “memory making” *(Mother of a 6yo *,*47yo*,* 1 child)* experiences. Enhanced by the reciprocal benefits of nature to parents and children, natural environments were described as places that promote family cohesion. Positive family processes included parent-child bonding through passing down of nature-based skills, knowledge and values, and shared enjoyment of nature-based activities. “It [time in nature] gives us an area of connection that we’re both interested in… when you’re out and about and creating memories, that helps… strengthen your bond as a parent and child” (*Mother of a 5yo*,* 30yo*,* 1 child*).

A commonly held view was that “parenting is easier outdoors”, and was attributed to a range of individual benefits for children and parents that were believed to facilitate positive dyadic processes and overall family harmony. Parents described differences in their children’s behaviors when “indoors” compared with “outdoors”. For example, while prolonged periods inside contributed to children seeming “cooped up”, opportunities for outdoor play in “open space” provided a “physical outlet” (*Mother of a 6yo*,* 35yo*,* 1 child*) and creative stimulation that was believed to promote positive sibling dynamics: “when they’re outside, they will go off and play for hours… imagination just goes wild… In the house, they play on top of each other… so there is a bit of conflict that can happen in that space” *(Father of a 6yo*,* 37*,* 3 children)*. Participants also reflected on how differences between indoor and outdoor environments influenced their experiences of parenting: “If we’re inside our house, he seems cooped up. And so that’s where his energy seems really big… when we’re outside it’s like, it’s just balanced. And so parenting’s easier. I’m happier, we’re both happier” *(Father of a 5yo*,* 41yo*,* 2 children)*. Following the shift to an outdoor environment, one parent described the reciprocal benefits to herself and her children:

“Often the amount of noise and things inside gets me frazzled… once we’re outside it feels a little bit less overwhelming for me… there’s space for them to move and space for me to breathe and think, and I find them a lot more able to play well by themselves or with each other outside… there’s a lot more conflict inside than there is outside. I use it probably more mindfully for the children, but I think it definitely works for me as well.” (*Mother of a 5yo*,* 33yo*,* 3 children*).

Parents believed that natural environments provide rich opportunities for children to “explore” their surroundings, stimulating curiosity, imagination, and creativity - leading to immersive, industrious, and autonomous play. Some parents reported they could “have a break” when their children were “occupied” playing in nature: “parenting is easier when we’re outside… there’s less involvement required because his moods different, he’s calmer… he’s really focused on the thing he’s doing” *(Father of a 5yo*,* 41yo*,* 2 children)*. Other times, nature-based activities were believed to promote pro-social behaviors and dyadic bonding:

“We have an orchard in the garden, so we’re out there, we’re working in the garden, and that’s kind of the environmental setting that I’m talking about when I say, connected in, or busy in nature … whether he’s holding onto a wheelbarrow helping us do tasks… that’s when he’s like, he’s locked in like he loves that task, and we’re all doing it together”. *(Father of a 5yo*,* 41yo*,* 2 children)*

Natural environments were described as having a “calming” effect on both children and parents, providing “a place where you are free from stress” (*Mother of a 7yo*,* 48yo*,* 1 child*) and opportunities to mentally “reset and recharge” (*Mother of an 8yo*,* 44yo*,* 2 children*): “it can distract her and take her mind off things… she’s not worrying about anything while she’s running around, it does take her out of herself and she just… she just focuses on being outside and enjoying herself” (*Mother of a 6yo*,* 42*,* 2 children*).

In contrast, parents believed that navigating children’s screen use was difficult, required effortful parental management, and strained family dynamics. Screen-time negotiations, often described as “tough”, “tricky”, and “frustrating”, were believed to result in parent-child conflict and feelings of parental stress and anxiety:

“There’s a lot of guilt over just even letting them use screens or the amount of time that they are allowed to have screens. And then when you get the push back, it’s complete frustration, then I often think I shouldn’t have even let her use it in the first place or… regret over not being firm enough”. *(Mother of a 7yo*,* 43*,* 4 children)*

Screen negotiations downgraded the perceived quality of parent-child exchanges: “making negotiations around the screen time… just kind of takes away the enjoyment from the interaction” (*Mother of a 5yo*,* 33yo*,* 3 children*). Parents also noted the impact of children’s screen fixations on parent-child communication, where children are “unaware of what’s happening” (*Mother of a 5yo*,* 30yo*,* 1 child*) around them during attempted interactions: “they are just so single focused once they’re on a screen, they often don’t hear you when you talk to them” *(Mother of a 7yo*,* 43*,* 4 children)*.

### Routines, expectations and negotiations

Participants believed that clear parenting expectations and family rules, combined with consistent routines, were associated with positive outcomes around both screen-based and nature-based family interactions. While clear and consistent rules around children’s screen use were believed to result in less screen-related negotiations and tensions because children “know the routine” *(Mother of a 7yo*,* 41yo*,* 2 children)*, flexibility around screen rules was often believed to result in more problematic behaviors and parent-child conflicts: “where the expectations weren’t clear it was often a battle when it was time to finish up… maybe he felt like he could push the boundaries a bit more because there weren’t those clear expectations in place” (*Mother of a 5yo*,* 34*,* 2 children*). 

Conflicting family views around children’s screen use was a commonly reported barrier to parental screen management, resulting in family conflict and challenging parental self-efficacy beliefs. In contrast, parents with shared values and a consistent approach to household screen rules communicated a sense of confidence around screen management: **“**the key is that we're both consistent in how we apply the different activities across the household …consistency is the best resource that we have” (*Father of a 6yo, 37, 3 children*).

Parents also believed that the benefits of nature to child and family wellbeing were strengthened through routine family experiences such as “weekend trips”, family camping holidays, or even repeated visits to “local parks”. Long-standing family rules and associated expectations that time in nature is “screen-free”, supported parents with screen management: “they don’t ask for it… cause they’ve grown up, they’ve been camping since they were babies and when we go camping there’s no screens” *(Mother of an 8yo*,* 44yo*,* 2 children)*. One parent commented that promoting screen-free routines during nature trips “makes clarifying boundaries a bit easier” (*Mother of a 5yo*,* 34*,* 2 children*).

### Nature provides relief from screen negotiations

Parents reported that children did not request screen time when in nature, where opportunities for immersive play enabled them to “disconnect from technology” and “switch off” from thinking about screens. Accordingly, family holidays in nature were described as opportunities for parents to “have a break” from screen negotiations along with other scheduled activities, responsibilities, and distractions. The combination of positive child play opportunities and less screen requests and negotiations provided parents with a sense of relief from screen-related parenting challenges and associated guilt:

“When we’re out in the bush… they are very good at creative play, occupying themselves… going finding the other children and creating their own games or activities… They don’t need the screens and because we haven’t created an option for them, we don’t get humbugged about it”. (*Mother of an 8yo*,* 44yo*,* 2 children*)

When discussing the home environment, parents reported that the availability of nearby outdoor spaces (e.g., garden or safe neighbourhood street) helped with screen management by providing children with opportunities for regular outdoor play as an alternative to screen time. Correspondingly, other parents perceived their residential layout as a barrier- “I think it would be easier [to manage child’s screen use] if we lived not on a main road… where the kids could play on the street or in the front yards” (*Mother of a 6yo*,* 35yo*,* 1 child*).

### Are parent and child interests compatible?

Parental beliefs about the benefits of screen use and nature exposure on wellbeing, were influenced by the perceived level of compatibility between child and parent activity preferences and values. While a shared enjoyment of screen-based or nature-based activities strengthened perceived relational benefits, incompatibilities between parent and child interests were perceived as a barrier to dyadic bonding. For example, one parent described the difficulties associated with a misfit between her own and her son’s outdoor interests:

“I really like doing things like bush walking, where it’s literally just nature - we’re just out there to look at the trees, try and find a koala, that kind of stuff. Jake is not big on that… he likes to be running, chasing a ball, or chasing friends… the tension’s already high [during a bushwalk] because I know that whinging is gonna accompany the activity.” (*Mother of a 6yo*,* 35yo*,* 1 child*).

One parent found a way to combine her own and her child’s interests to enhance her enjoyment of outdoor family time despite divergent outdoor activity preferences:

“being able to do something I enjoy as well as be there with them, so if they’re out playing, I can just sit in the garden and do something that I enjoy, and then go be with them and do something else. So, then I’m not feeling like I’m sitting there doing nothing. I find that helps me a lot.” (*Mother of a 5yo*,* 41yo*,* 2 children*).

Similarly, while a shared enjoyment of screen-based activities was perceived as a “bonding” experience for some parents, incompatibilities between parent and child screen views were perceived as a barrier to quality time, as illustrated by one parent’s attempts at playing video games with her son: “I don’t like it at all, I don’t feel good playing it and, I don’t see how he can enjoy it” (*Mother of a 6yo*,* 35yo*,* 1 child*).

### From contented parenting to parenting concerns

This theme explores the spectrum of parental experiences ranging from feelings of contentment when children’s routines are balanced - supporting their overall wellbeing - to concerns about maintaining this balance as children develop greater autonomy around screen-related behaviors.

Parents described wellbeing as a multi-dimensional construct, with different facets of physical, emotional, social and mental health “balanced” and “in alignment”. This value underpinned perspectives around children’s screen use and nature exposure, as well as parent motivations behind facilitating or curbing these activities. Although parents recognized positive screen uses, overarching beliefs were contextualized within broader concerns around the impacts of screen use on children’s overall wellbeing and the balance of family dynamics. While parents believed that digital literacy was important for children’s overall development, others chose to delay screen access due to the perceived benefits: “he may be missing out, but hopefully not too much… I think the benefits outweigh the lack of learning of how to use a screen” (*Mother of a 7yo*,* 48yo*,* 1 child*).

A commonly held view was that screen time is superfluous to children’s overall wellbeing, when opportunities for entertainment, education and social connection could be sourced elsewhere without the potential risks. Contrastingly, when asked “what is associated with wellbeing”, most parents shared the belief that outdoor time is beneficial across multiple dimensions of health: “[outdoor places are] a mix of time spent with the girls, a chance for them to play together, a chance to run around and use their bodies, to explore and jump around” (*Mother of a 6yo*,* 42*,* 2 children*).

Parents described a range of child activities as “consuming”, however accounts were imbued with contrasting meanings for screen-based and non-screen-based activities. Immersion in nature-based activities was perceived as a “healthy” passion, with parents reporting a sense of contentment with unrestricted timeframes around child-led outdoor play. In contrast, screen fixations were labelled as “addictive” (see theme 1.2.2), with prolonged screen time perceived as a threat to children’s physical (e.g., impacts on eyesight), social, and mental health (e.g., cyber safety concerns and anxiety). Although most children engaged in a healthy variety of activities, parents felt that without parent-led screen controls, their children’s wellbeing balance would be threatened due to the combination of dominant screen preferences (theme 1.1), persuasive digital environment (theme 1.2) and children’s age-related vulnerabilities (e.g., development of self-regulation and impulse control).

Screen time balance was achieved differently across families. For some parents, a mindful approach to screen content and context (e.g., co-viewing and scaffolded content versus “zombying out” and unrestricted content) was associated with a sense of parenting confidence. Other parents balanced their children’s screen time with outdoor time: “to try and get the balance, some TV is okay, as long as it’s balanced with an appropriate amount of time doing other things outside” (*Father of a 6yo*,* 37*,* 3 children*).

Appraisals of child activities were further contextualized within an understanding of how unique child needs and characteristics (i.e., disposition and temperament) interact with the needs of the dynamic family unit. For one parent whose focus was finding harmony between the needs of neurodivergent family members in a home-schooling context, screen-related “down time” was perceived as an integral part of a balanced family life.

While parents perceived nature-based experiences as opportunities to help children form health-positive habits that may extend into older childhood and adulthood, they were concerned about the interaction between growing child autonomy and child preferences for screen-based activities. These prospective concerns were informed by both lived experiences (e.g., problematic screen behaviors), social observation (e.g., screen reliance), and external sources of information (e.g., knowledge acquisition through parenting blogs). Parents were primarily worried about cyber safety risks and the potential impacts of maladaptive screen habits on their children’s mental health during older childhood and adolescence.

Parents emphasized their role in shaping their children’s routines to influence health-related behaviors. For example, as screen time was a perceived barrier to children’s outdoor time, parents described their role in “providing the scaffold” (*Mother of an 8yo*,* 44yo*,* 2 children*) to help children overcome this barrier (for example parental guidance during difficult transitions away from screen time):

“To make the transition from TV to getting outside. It’s a conflict. It’s a fight… he’ll resist you with that first step, but once you get him out… once he’s made that switch in his mind - he’s happy, and he goes off… guiding him through that transition can be a challenge at some point.” (*Father of a 6yo*,* 37*,* 3 children*).

Another parent described the shift in her child’s attitude toward outdoor time following the transition away from screen time “Leah is like - I don’t like the sand, and I don’t like the beach, but when she’s there she has fun” (*Mother of a 7yo*,* 49yo*,* 1 child*).

Parents believed outdoor routines could help their children find ways to balance screen time in later life: “I hope that he’ll want to continue doing that [outdoor time] when he’s older, and can make his own choices, that he’ll actually choose to be outside rather than hanging out in front of a screen” (*Mother of a 5yo*,* 34*,* 2 children*). However, many parents were also apprehensive about the combination of their diminishing influence over their child’s screen use habits and impending screen-related challenges:

“I think that’s probably the reason that I find them [screens] concerning, is I don’t feel like there’s much you can do, it’s part of them growing up and you having to just kind of let go and hope that the values and things that you’ve instilled and relationships you’ve cultivated is enough to get them making good decisions on their own”. (*Mother of a 5yo*,* 33yo*,* 3 children*)

## Discussion

This study contributes novel perspectives to existing empirical knowledge by exploring, from parents’ perspectives, relationships between children’s screen use and outdoor time as two interacting determinants of health. Parents are centrally positioned within a child’s development and their beliefs and perceptions represent a mosaic of lived family experiences and observations. As such, parent accounts can provide holistic and nuanced understandings around how individual child characteristics interact with a multitude of parent, family-level, societal, and environmental factors to shape children’s health behaviors. Acknowledging these dynamic interactions, findings have been interpreted through the lens of two contemporary theoretical frameworks that ground children’s screen use [18] and nature exposure [31] within relational developmental systems.

### Wellbeing balance, parental self-efficacy, and children’s problematic media use

Findings contextualize prior research investigating associations between excessive screen use and children’s biopsychosocial health [1, 2] and mirror larger-scale data identifying children’s screen use as the leading concern for Australian parents [20]. Framed by the interactional theory of childhood problematic media use [18], in-depth accounts illuminate some of the mechanisms underlying parental screen concerns. Notably, findings illustrate the complex and reciprocal relationships between parental self-efficacy, the role of the digital environment, screen-related child behaviors and child engagement with other health-promoting activities.

Parent accounts demonstrate the bidirectional relationship between parental self-efficacy beliefs and perceived screen-related challenges identified by the interactional theory of childhood problematic media use [18]. For example, parents who reported challenging screen-related child behaviors (e.g., persistent screen time requests, “tantrums” and difficult transitions away from screen time) often felt defeated or apprehensive in their attempts to navigate screen-related challenges. Low perceived parental self-efficacy was in turn associated with reports of inconsistent parental screen limiting practices and difficult screen-related behaviors. Similarly, significant associations between inconsistent parental screen-management and problematic child screen use have been observed in prior research [39]. Conversely, parents who communicated a high level of parental self-efficacy held more optimistic views around screen management.

Interestingly, parents communicated positive screen-related parental self-efficacy beliefs when their child’s screen time was balanced with outdoor time. This was attributed to the role of outdoor time in promoting “balance” between children’s physical, social and psychological health – an overarching wellbeing value reported by parents. Conversely, while parents recognized positive screen uses, they were concerned about the impact of screen time on children’s wellbeing balance. This was commonly attributed to the persuasive influence of the digital environment on child behavior and free time preferences.

The interactional theory of childhood problematic media use [18] identifies the role of technology as a distal factor that interacts with other proximal (e.g., parental self-efficacy) and maintaining factors (e.g., peer screen engagement) to perpetuate problematic child screen use. According to the model, curated digital content tailored to individual child needs and persuasive screen features (such as fast-paced content) are designed to capture attention and prolong screen use - perpetuating maladaptive screen-related child behaviors. In discussing concerns around the persuasive nature of screen devices, parent accounts flesh out some of the hypothetical pathways between the “engagement-promoting design features” [18] of modern technologies and problematic child screen use. For example, parents believed that screen activities often resulted in a state of “fixation”, leading to dysregulated child reactions to screen time rules, disruptions in parent-child communication, and difficult screen transitions. These behaviors corroded parental self-efficacy beliefs, consequently challenging parental screen management. Parental decisions to delay child screen access reflect this experience – many parents previously “trialled” screen time and found that associated behavioral and relational challenges outweighed the benefits. Congruent with recent empirical work [3], findings suggest that the digital environment may directly influence child behavior, its role in problematic child media use warranting greater research attention.

Findings also provide novel insights around unexplored interactions within Domoff, Borgen and Radesky’s model [18], such as whether unique child-level characteristics and vulnerabilities interact with the digital environment and free time preferences to displace regulatory behaviors such as outdoor time. Parent accounts suggest that child predispositions (e.g., self-regulation difficulties) do interact with persuasive digital environments and screen-based activity preferences, resulting in a reduced interest for other health-promoting activities. As identified in prior research [40], children’s screen preferences and inclinations were perceived as a barrier to nature exposure. However, parents played an instrumental role in helping children overcome this barrier. Whether child screen preferences reportedly led to actual screen behaviors that displaced health-promoting activities, was largely influenced by perceived parental self-efficacy. For example, parents with a high sense of parental self-efficacy reported that despite behavioral resistance, they felt effective in helping their children transition to other activities such as playing in the garden. For others, low outdoor self-efficacy interacted with child screen preoccupations and peer influence around screen activities - leading to reports of inconsistent parental screen limiting practices.

### Pathways between nature exposure and family cohesion

This research recognizes nature exposure as conceptually nestled within the construct of outdoor time. Hence, both constructs (as reported by parents) are presented and discussed in relation to key themes. Although parents discussed general “outdoor time” and nature exposure interchangeably, outdoor “open space” often accompanied descriptions of increased physical activity and the positive outlet this can provide for children’s “excessive” energy. However, outdoor environments characterized by features of nature (e.g., forests or vegetated backyards) were associated with perceived mental restoration for children and parents, pro-social child behaviors and positive play behaviors (e.g., creative interactions or industrious independent play with sustained attention). Importantly, compared to other settings (e.g., indoor spaces), environments characterized by nature were associated with positive family processes and family cohesion – as seen in prior research [32, 33].

As proposed by the integrative model of family-based nature activities [31], family activities in nature promote family cohesion through a combination of individual psychological restoration [29] and positive relational processes. Firstly, current findings illustrate the role of Attention Restoration Theory’s central tenets (activity compatibility, opportunity for immersion, and a feeling of being ‘away’ from daily stressors) when families spend time in nature. For example, while incongruent outdoor preferences between parents and children were perceived as a barrier to nature connection, shared enjoyment of nature-based activities was associated with positive child behaviors, mental and emotional wellbeing for parents, and parent-child bonding. Parents also reported that time in nature helps them “switch off” from everyday pressures, distractions, and responsibilities. Likewise for children, natural environments provided opportunities to “disconnect” from screen-related distractions through immersive play. Although the mentally restorative role of nature in negating perceived ‘technostress’ has been explored by occupational research [41], current findings illustrate this association within a family context.

Present findings support Izenstark and Ebata’s [31] assertion that “family rituals in nature create symbolic and meaningful interactions that influence family functioning”. Family traditions such as camping trips or regular visits to nearby nature were associated with positive family rituals that were described as enjoyable and “memory making” for both parents and children – reportedly leading to bonding behaviors and cohesiveness amongst family members. By focusing on family processes and dynamics, findings illuminate why the ‘relational dimensions of nature’ described within the field of environmental psychology may be more significant predictors of wellbeing than proximity to green spaces [30]. Parent accounts revealed that the perceived benefits of nature can be derived from a combination of repeated family experiences in nature, the opportunity to disconnect from screen-related distractions and relational challenges, and positive interpersonal processes.

### Reciprocal parenting concerns and motivations

Congruent with prior research [9], patterns across findings illustrate that parents perceive nature exposure and excessive screen time as inversely associated with children’s psychosocial health. Findings build on prior research by illustrating that the relational challenges parents associate with child screen use also mirror the reported interpersonal benefits of family-based nature experiences. Correspondingly, reciprocal parenting motivations underpinned decisions to either facilitate or restrict nature-based and screen-based activities. Some parents described engaging with nature more broadly as a way of maintaining wellbeing balance for their children, while others sought family-based nature experiences to directly support them with screen-related challenges. For example, some parents noted that children display more positive social and play behaviors when outdoors compared to indoors, and others directly sought nature-based opportunities to counteract relational challenges associated with children’s screen use. While child behaviors such as frequent requests for screen time and difficult transitions from screen-based activities were often associated with parental guilt and perceived as a challenge to parental self-efficacy, parents reported that “parenting is easier outside”. Previous qualitative research has generated similar findings – for example, a study investigating mother-daughter experiences in nature generated the theme ‘got along better outdoors’ to represent perceived improvements in dyadic processes when outdoors compared to indoors [42]. Authors attributed these relational improvements to increased opportunities for quality bonding behaviors, improved psychological wellbeing and reduced need for “parental policing”. Our findings support and extend this reasoning: family-based experiences in nature were often seen as opportunities for the family to disconnect from technology so parents could have a break from effortful screen-related negotiations and family conflicts, and children could experience a mental “reset” from the distractions of technology.

### Integrating two models: deepening our understanding of children’s health behavior

#### Family-based nature activities: a protective factor for problematic child media use

**Fig. 2:**
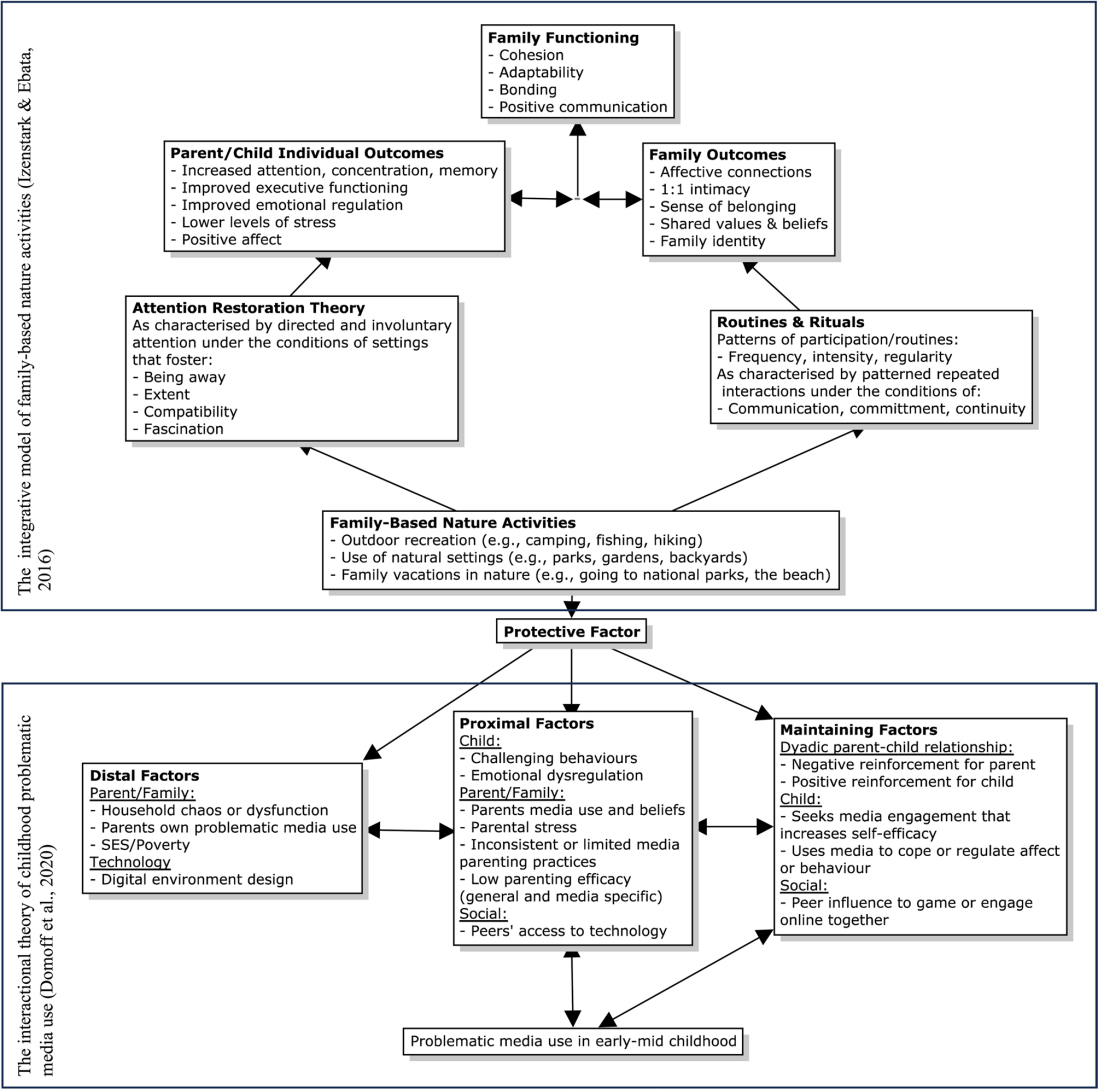
Family Nature Activities as Protective Factor for Problematic Child Screen Use

As illustrated in Fig. 2, findings suggest that family-based nature activities may simultaneously act on multiple factors within the interactional theory of childhood problematic media use [18]. These include distal factors such as household dynamics and parents' own media use, proximal factors such as children’s emotional and behavioral regulation, parental self-efficacy, parental media beliefs and screen limiting practices, and maintaining factors such as peer influence toward online engagement and parental screen-provision to regulate behavior or occupy the child.

The important role that protective factors play in the etiology of children’s behavioral disorders is recognized by central developmental child theories [16] informing the interactional theory of childhood problematic media use [18]. However, while emphasis is placed on the interacting spheres of influence that may increase the risk of problematic child screen use, the role of protective factors remains underexplored. Findings from this study suggest that family-based experiences in nature may serve as an important protective factor that may decrease the risk of problematic child screen use. For example, the model identifies low parental self-efficacy (both general and media-specific) as a proximal factor contributing to problematic child screen use. Present findings provide novel and meaningful examples of how parental self-efficacy around outdoor and nature-based family experiences may interact with the model by indirectly influencing screen-related child behaviors.

Current preventative approaches to child health are largely fragmented in their approach. For example, nature-based research focuses on individual socioemotional child outcomes while screen time research focuses on physical activity and sedentary behaviors [14]. From a preventative perspective, understanding how children’s screen use and nature exposure interact within the family system can help identify practicable targets for intervention. Thus, theoretically informed by the integrative model of family-based nature activities [31] and supported by our data, we propose family-based nature activities as an added protective factor for the interactional theory of childhood problematic media use [18].

### Children’s screen use: potential moderator to family-based nature experiences

This research also demonstrates how more nuanced perspectives around children’s screen use can strengthen our understanding of other health-related child behaviors such as nature-based activities. Findings suggest that children’s screen use can act as a potential moderator in the integrative model of family-based nature activities [31] by acting as either a barrier or facilitator to children’s nature connectedness and nature exposure. In-depth parent accounts illustrate that the influence of screen use on children’s engagement with nature is not binary, but rather fluid and context-sensitive – as represented by themes 2 and 3. For example, dyadic conflicts around children’s screen use may interfere with positive relational outcomes derived from nature-based experiences. Conversely, screen-based activities and applications can support family-based nature activities by acting as ‘features of the setting that influence feelings of comfort and safety’ [31]. For example, a parent’s sense of safety can be enhanced through specific screen applications (means for emergency contact and satellite navigation) while screen-based learning opportunities can facilitate children’s nature connectedness. Findings also provided some preliminary evidence for how this relationship may play out for families with neurodivergent children (e.g., for one child diagnosed with Autism, using a tablet in natural settings helped transition from solo indoor play to interactive outdoor nature-based activities).

### Recommendations and future directions

#### Beyond displacement of physical activity and the future of child health behavior guidelines

National child health guidelines focus on the benefits of outdoor time as a function of physical activity that displaces indoor sedentary screen time [5]. However, beyond this time-use relationship, current findings explore meaningful mechanisms between children’s screen use and outdoor time that map onto a multitude of other child health behaviors. For example, parent accounts suggest that children’s screen-based activity preferences can act as a barrier to dyadic parent-child processes, health-positive play behaviors, and nature connection (as demonstrated in prior research [40]). Conversely, time in nature can provide experiences that are mentally restorative for parent and child and promote family cohesion through positive relational processes – potentially reducing the risk factors associated with excessive child screen use. From this perspective, the distribution of child health behaviors associated with children’s screen use and outdoor time has broader implications on family wellbeing and child development beyond physical health. Further research is needed to explore these mechanisms quantitatively, to stimulate more integrated, evidence-based perspectives to inform child health guidelines.

The rich qualitative data informing this study emphasizes that screen-related child outcomes are sensitive to both screen-contexts and relational dynamics within unique family systems. For example, many parents were cognisant of unique differences between screen-based media type and context (e.g., scaffolded co-viewing was perceived “bonding” while unsupervised and unrestricted screen time was often perceived as unhealthy screen fixations). Importantly, parent views around children’s screen use were informed by temporally observed patterns of child behavior, dynamic relational processes and interactions with overall child and family wellbeing. Rather than a narrow outcomes-driven approach, as is common within screen time research (i.e., the influence of specific screen-based learning apps on children’s cognitive outcomes [43]), parental screen-related appraisals considered the interplay of broader child health behaviors and relational outcomes. For example, parental screen concerns were shaped by prior experiences with problematic screen-related child behaviors resulting in difficult family dynamics, and screen-based activities replacing health-positive play behaviors. Despite the importance of contextualizing children’s screen use within the dynamic, relational family paradigm, national child health guidelines focus on unidimensional, time-based measures of children’s screen use. Likewise, an exclusive focus on the material dimensions of outdoor time (e.g., proximity to green spaces) is insufficient for a comprehensive understanding of how children’s outdoor time influences wellbeing outcomes.

Lastly, parent accounts reflect limitations around negatively geared, screen limiting messaging [5, 6] that may result in feelings of parental guilt [3] and unintentionally weaken effective parental screen-management. When parents were asked what would support them with screen-related concerns, many were “unsure” about possible solutions and supports (often after unsuccessfully trialling screen time limits) or stated that parents “only ever hear the bad things [about screen time]” *(Mother of a 7yo*,* 48yo*,* 1 child)*. Although parents commonly described time in nature and screen use as two discrete variables inversely associated with child and family wellbeing, few explicitly identified nature exposure as a screen-management strategy. Existing approaches to parental screen-awareness can be strengthened by dually addressing the related constructs of screen use and children’s engagement with nature with a focus on health-positive messaging that strengthens parental self-efficacy beliefs.

#### Transitions to autonomy and opportunities for parental intervention

Together with a sense of responsibility for managing their children’s screen use, parents shared prospective concerns around the role of technology (the persuasive digital environment and child screen preoccupations) during growing child autonomy, when the parent’s role diminishes. This included concerns around potential mental health impacts of unsafe cyber practices and maladaptive screen-related habits in later life. As parent-focused interventions may be more effective than child-focused interventions for the management of problematic screen use [7], middle childhood represents an opportune time for parental intervention. Longitudinal research may help understand whether parental interventions dually focusing on screen use and nature exposure can result in the formation of longer-lasting health-positive habits as children gain autonomy in adolescence and later life.

### Limitations

Qualitative research aims to describe participants’ contextually bound and subjective lived experiences through rich, detailed accounts, rather than striving for generalizability or broad transferability of findings. Therefore, while the descriptions reflect both commonly held views and variations across participants’ beliefs and perceptions, the findings are specific to the socio-cultural context of the study (e.g., social norms surrounding screen use or location-specific barriers to accessing nature). Although the purposive sampling matrix guided the distribution of family compositions and socio-economic backgrounds, the sample lacked cultural diversity, gender distribution favoured females and most participants were from higher income families. Secondly, although interviewers were mindful of avoiding value judgements, internalized parenting guilt or perceptions of judgement may have led participants to provide more socially desirable answers. Moreover, parents with higher parental self-efficacy may have been more confident to respond to recruitment efforts – potentially influencing the diversity of parent views.

## Conclusion

Parent perspectives provide valuable insights around how children’s health trajectories are shaped by a multitude of parent, child-level and societal factors that interact within the relational family system. By exploring parent accounts through the lens of two integrated relational models [18, 31], we propose that family-based nature activities may act as an important protective factor for the etiology of children’s problematic screen use. By exploring themes-as-continuums, findings emphasize the nuanced bidirectionality of relationships and the role of contextual factors - challenging binary views around children’s screen time and outdoor time. The present research can inform the timely development of integrated parenting support strategies that dually address biopsychosocial wellbeing for children and parents, while simultaneously strengthening relational family outcomes.

## Supplementary Information

Supplementary Material 1.

Supplementary Material 2.

Supplementary Material 3.

## Data Availability

Due to the nature of qualitative interviews, the data are not publicly available however can be made available upon request from the corresponding author [MT].
