# Supplementary material for: Nuances of balance; parental perspectives around screen use and nature exposure in middle childhood
Source: BMC Psychol. 2025 Dec 22;13:1398. doi: 10.1186/s40359-025-03552-9 (PMC12752391; doi:10.1186/s40359-025-03552-9)
Supplement: Supplementary file 2 — Supplementary Material 2. [file 40359_2025_3552_MOESM2_ESM.docx]

**Nuances of Balance; Parental Perspectives Around Screen Use and Nature Exposure in Middle Childhood**

**Online Resource 2: Qualitative Interview Script**

**Starting Interview**

1. [Introduce yourself and make general conversation, e.g., “how has your day been so far?”, etc.]

2. [Reassure the participant that they can answer in any way they want and there is no right or wrong way to answer a question, and no judgment on their responses]

3. [Remind them that the interview will be recorded and that any identifying information will be removed or replaced during transcription]

**Interview script**

Hi [participants name], thanks so much for your time today, how are you doing/ how’s your day been so far?

I’m [name] and I am a researcher in this project run through [name of University]. We really appreciate you taking the time to contribute to an important and new area of research. As we discuss your thoughts today, I would like to emphasise that are no right or wrong answers and that you are free to answer the questions in any way you like. The interview will take anywhere between 20 - 30 min but if at any point, you want to take a break or stop, please feel free to let me know.

I would also like to have a plan for any technical issues we might encounter. If our zoom meeting cuts out for any reason, would you be ok for me to give you a call on the mobile number you have provided?

As you would have read in the PLS we sent through, the interview will be recorded, and the recording will afterwards be transcribed. Your name, your child’s name and any other identifying information will be either removed or replaced with pseudonyms. Does that all sound ok to you? Do you have any questions before we begin?

I’m going to start recording now.”

***START RECORDING***

[Questions numbered (a), (b).. to be asked, bullet points to only be used as prompts if necessary]

**1. Background on Child and Household**

Thanks once again for taking part in our previous online survey, we can see that the child you had in mind to talk about during this interview is [age of child], could you tell me [his/her] name?

To start off, can you tell me a little bit more about [child’s name]?

As you know, we’re interested in hearing about parent’s perspectives around screen use and time in nature. Some of the questions will be general questions about family activities and dynamics, while other questions will be more specific to your experience with [child’s name].

**2. Perceptions around family wellbeing**

1. What does wellbeing mean for you?

- What about your child’s/your/your families wellbeing?
- What do you think is associated with wellbeing?

**3. Parenting challenges**

1. Do you have any specific concerns about your child’s wellbeing and development?
2. How do these concerns/child behaviours affect your relationship with your child?

- [if child behaviour is **not** mentioned]: What about in regard to your child’s behaviour specifically?
- What about any general concerns for your child as they grow up?

Ask the questions in the below box **ONLY** if **screen time** or **nature** is ***mentioned*.**

| - How do you feel when your child... [restate the child behaviour mentioned by participant]? (e.g. 'You mentioned that when (child's name) uses screens they can become a bit (behaviour), can you describe that a bit more?') - What sorts of things do you do to manage these behaviours? - How does your child respond when [insert the strategy mentioned by parent]? - [IF parent mentions ‘outdoor time’]: can you describe the kinds of outdoor environments you are talking about? |
| --- |

1. In an ideal world, what would help support you with these concerns? [can specify/reinstate previously mentioned concerns/behaviours if needed]

**4. Perceptions around existing family routines**

1. What does your child enjoy doing in their free time?

- [IF ST/NE related answers]: How do you feel about your child spending time [… e.g on screen devices, playing outdoors].

1. What does quality family time look like to you?

- Are there particular places you like to go with your child/ren?
- [IF NE related answers (e.g time outdoors): what does outdoor time mean to you?

**5. Safety net questions:** [Only ask if participant has **NOT** discussed **screen use** and **nature exposure**.]

We are also interested in gathering information about parents’ perspectives around their children’s screen use and time in nature, so I’ll ask some specific questions about those topics.

At the beginning of the survey, I asked about your thoughts regarding your child’s wellbeing.

(g) How do you think **screen use** interacts with your child’s and your family’s wellbeing?
AND/OR

(h) How do you think **nature-exposure** interacts with your child’s and your family’s wellbeing?

- How does time in nature influence your relationship with your child/ren?
- How do you feel when you spend time in nature with your child/ren?

(i). What does family time in nature look like to you?

**6. Other targeted ST/NE questions:**

[read intro if safety net questions NOT asked]: We are also interested in gathering information about parents’ perspectives around their children’s screen use and time in nature, so I’ll ask some specific questions about those topics.

1. Does your child have access to screen-media devices when out in nature?
2. Does your child ask to use screen media devices when out in nature?
3. **Conclusion**

And that brings us to the end of the formal interview questions, is there anything else that you think is important that you’d like to talk about today?

**Post-interview**

So that wraps up the interview. Thanks so much for taking part in this interview, we know how busy it is being a parent and your time is greatly appreciated. We will be in touch via email when we’ve transcribed your data with a copy of the transcript as well. You can go through it if you’d like and let us know if you’d like to change or remove anything you have said during the interview. Do you have any questions? Thank you again, have a great rest of the day!
