# Supplementary material for: Nuances of balance; parental perspectives around screen use and nature exposure in middle childhood
Source: BMC Psychol. 2025 Dec 22;13:1398. doi: 10.1186/s40359-025-03552-9 (PMC12752391; doi:10.1186/s40359-025-03552-9)
Supplement: Supplementary file 3 — Supplementary Material 3. [file 40359_2025_3552_MOESM3_ESM.docx]

**Nuances of Balance; Parental Perspectives Around Screen Use and Nature Exposure in Middle Childhood**

**Online Resource 3: A-priori Coding Template**

| *Category 1* | *Category 2* | *Category 3* |
| --- | --- | --- |
| Features | NE material dimensions | Proximity/ quality |
|  | Digital Environment | Device type/content |
|  | Home environment | Household layout & characteristics |
| Context | NE relational dimensions | Nature connectedness |
|  |  | FBNA (what/how: type) |
|  |  | FBNA (who: social context) |
|  | Screen use contexts | SU context/reasons |
|  |  | SU content |
| Parent | Values, appraisals, beliefs | Sources of information |
|  |  | Parenting concerns (current and future) |
|  | Parental self-efficacy | Barriers & facilitators |
|  | Individual characteristics & circumstances | Parent-level barriers |
|  |  | Individual free time preferences |
|  |  | Parents own media use/ media-related behaviours |
|  |  | Nature: attention restoration |
| Child | Individual characteristics | Temperament |
|  |  | Typically/non-typically developing |
|  |  | Free time preferences |
|  | Individual outcomes | Nature: attention restoration |
|  |  | Behaviour |
| Social | Norms & external influences | Groups of belonging/culture |
|  |  | Perceived social norms/social comparison |
|  |  | Learning through social observation |
| Family Functioning | Individual family contexts | Unique family composition |
|  |  | Family routines |
|  | Dyadic interactions | Communication, conflict, quality time, bonding behaviors |

*NE: nature exposure, SU: screen use, FBNA: family-based nature activities
